# Plant immunity: Rice XA21-mediated resistance to bacterial infection

**DOI:** 10.1073/pnas.2121568119

**Published:** 2022-02-07

**Authors:** María Florencia Ercoli, Dee Dee Luu, Ellen Youngsoo Rim, Alexandra Shigenaga, Artur Teixeira de Araujo, Mawsheng Chern, Rashmi Jain, Randy Ruan, Anna Joe, Valley Stewart, Pamela Ronald

**Affiliations:** ^a^Department of Plant Pathology, University of California, Davis, CA 95616;; ^b^The Genome Center, University of California, Davis, CA 95616;; ^c^Department of Microbiology and Molecular Genetics, University of California, Davis 95616, CA

**Keywords:** XA21, raxX, resistance, sulfotyrosine, gram-negative bacteria

## Abstract

In this article, we describe the development of the plant immunity field, starting with efforts to understand the genetic basis for disease resistance, which ∼30 y ago led to the discovery of diverse classes of immune receptors that recognize and respond to infectious microbes. We focus on knowledge gained from studies of the rice XA21 immune receptor that recognizes RaxX (required for activation of XA21 mediated immunity X), a sulfated microbial peptide secreted by the gram-negative bacterium *Xanthomonas oryzae* pv. *oryzae.* XA21 is representative of a large class of plant and animal immune receptors that recognize and respond to conserved microbial molecules. We highlight the complexity of this large class of receptors in plants, discuss a possible role for RaxX in *Xanthomonas* biology, and draw attention to the important role of sulfotyrosine in mediating receptor–ligand interactions.

## Significance

The mechanisms plants employ to resist infection were unknown until just a few decades ago. We now understand that plants utilize diverse classes of immune receptors to recognize and respond to pathogenic microbes and pests. This paper describes the development of the plant immunity field, from early studies on the genetics of disease resistance to our increasing knowledge of how plant receptors interact with their microbial ligands, with an emphasis on the rice immune receptor XA21 and its bacterial ligand.

Perception of extracellular signals by cell-surface receptors is of central importance to eukaryotic development and immunity. For example, in the absence of an adaptive immune system, plants rely on a repertoire of innate immune receptors to recognize potential pathogens and initiate defensive responses. A key research focus of the P.R. laboratory is to understand the principles and mechanisms that underlie the processes governing the immune response.

Here we describe our 30-y effort to dissect the genetic and molecular basis of the innate immune response in the staple food crop and model organism rice *Oryza sativa*.

## History of Infectious Disease and Plant Breeding

In 1845, potato farmers began losing much of their potato crop to a disease that later became known as late blight. This led to the Irish potato famine that has been linked to as many as a million deaths and launched the first serious investigations into the basis of infectious disease (1). In 1853, the German plant pathologist Anton de Bary identified *Phytophthora infestans* as the causal organism of late blight of potato. He demonstrated that *P. infestans* grows only from its own spores and cannot arise de novo, thus refuting the theory of spontaneous generation, popular at that time (2, 3).

In 1859, the French microbiologist Louis Pasteur showed that the spoilage of beer and wine results from contamination by bacteria introduced during the fermentation process (4). He knew of de Bary’s work and hypothesized that diseases of humans and animals also result from microbial infestation. However, it was not until 1876 that the causal role of microorganisms in animal diseases was demonstrated conclusively. This work was carried out by Robert Koch, who studied anthrax infection of cattle, using the mouse as a model host (5). Koch’s postulates, developed during these studies to establish a microorganism as a causal agent for a disease, applied equally to work with plant and animal pathogens thereafter.

These early studies set the stage for research by British geneticist and plant breeder Rowland Biffen (6–8). In 1905 he demonstrated that resistance to yellow rust in wheat is transmitted in a Mendelian fashion. He cross-pollinated a resistant wheat variety with a susceptible wheat variety and showed that the resulting seed carried the resistance of the parent. E. C. Stakman further showed that a gene conferring resistance against one “form” of the pathogen did not work against all forms of the pathogen and, in fact, that several pathogen types could coexist (9, 10). Today, more than 100 y after Biffen’s discovery, plant breeders have introduced “resistance genes” into virtually every crop plant that we consume. Despite the success of breeders and the widespread planting of resistant varieties, it was not until the 1990s that researchers finally uncovered the molecular basis of disease resistance.

## Flor’s “Gene-for-Gene” Hypothesis and the Isolation of Plant Resistance Genes

The current model for plant resistance, that a plant receptor interacts directly or indirectly with a microbial molecule, follows from genetic analyses conducted throughout the 20th century. These studies demonstrated that plants contain numerous resistance genes, each specific for a particular pathogen race encoding the molecule recognized by the receptor. This “gene-for-gene” model predicts that resistance results from positive contributions from both the plant receptor and a microbial molecule that serves as its ligand (11, 12). Although Harold Henry Flor named these hypothetical microbial molecules “avirulence” proteins, these pathogen-produced molecules were later renamed depending on the biological system as described below.

## A Time of Remarkable Discoveries: Cloning of the First Disease Resistance Genes

Loci conferring disease resistance have been identified in most crop species. Because scientists envisioned that isolation of a disease resistance gene would open the door to analyzing and ultimately understanding the molecular basis of plant defense against pathogen invasion, considerable effort was directed toward cloning genes conferring resistance to a variety of bacterial, fungal, and viral infections (13).

**Fig. 1.**
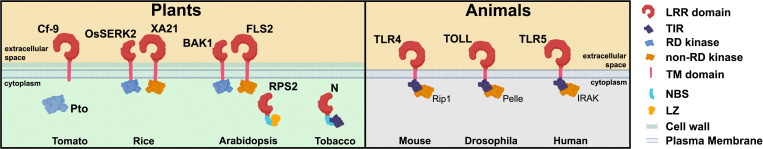
Immune receptor structures. Cell-surface immune receptors and coreceptors in plants and animals carry LRR domains (red). Plant receptors and coreceptors carry TM domains and kinase domains. Animal receptors associate with adaptor proteins and kinases via the TIR domain (blue). Kinases and kinase domains that carry the “non-RD” motif are colored orange. The RD kinase PTO is colored blue. NBS-LRR proteins contain LRRs and a TIR or Leucine zipper (LZ) domain (bright orange in RPS2). The detoxification enzyme HM1 is not shown.

In the 1990s, laboratories around the world made dramatic discoveries, using genetic approaches to isolate the first putative immune genes. These fell roughly into five classes based on their structure and predicted function (Fig. 1). These include a gene encoding a detoxifying enzyme, an intracellular kinase, intracellular receptors, cell-surface receptors, and cell-surface receptor kinases. For example, the maize gene *HM1* confers race-specific resistance to the fungal pathogen *Cochliobolus carbonum* (13). *HM1* encodes a NADPH-dependent HC toxin reductase, which inactivates the HC toxin produced by the fungus. In 1994, Gregory Martin’s group isolated the tomato *Pto* gene (14), encoding a serine threonine protein kinase that confers resistance to *Pseudomonas syringae* pv. *tomato* (*Pst*) strains expressing the gene *avrPto* (15). The third and largest group of resistance genes were isolated from *Arabidopsis* (RPS2 and RPM1), tobacco (N), and flax (L6) (16–18). These proteins contain leucine-rich repeats (LRRs), putative cytoplasmic signaling domains and nucleotide binding sites (NBS). Of particular importance was the observation by Barbara Baker’s laboratory that the tobacco N gene (17), which confers resistance to tobacco mosaic virus, shows similarity to the *Drosophila* TOLL protein isolated by the team of Kathryn Anderson (19). The N protein also shares similarity in its cytoplasmic TOLL/interleukin-1 receptor domain (TIR) with the interleukin-1 (IL-1) receptor in mammals and the TOLL protein. These NBS-LRR genes, which control resistance to three widely different pathogen types, are the foundation of a class of plant disease resistance genes that have been described in several excellent reviews (20, 21). The fourth class includes the tomato *Cf* genes (22) which encode LRR receptor-like proteins (RLPs) conferring resistance to *Cladosporium fulvum*.

The fifth class of disease resistance genes is represented by the rice *Xa21* gene, conferring resistance to the gram-negative bacterium *Xanthomonas oryzae* pv. *oryzae* (*Xoo*) (23). Compared with previously cloned genes, the structure of the XA21 protein represented a new class of plant disease resistance genes encoding a receptor-like kinase (RLK) (Fig. 1).

## Why Rice?

Research over many decades has shown that studies of a wide range of model species are needed to elucidate fundamental biological processes relevant to animals, plants, and microbes. In addition to the discovery of immune receptors described above, plant scientists have made numerous other discoveries relevant to animals: Mendel, McClintock, Cashmore, and Beijerinck discovered the laws of inheritance, transposable elements (24), circadian clock genes (25), and the first virus (26), respectively.

The P.R. laboratory chose to study rice, because it is a staple food for more than half the world’s people and because it is a model for studies of other monocotyledonous species, which includes the grains corn, wheat, barley, and oat. The rice variety Kitaake has emerged as a key model for genetic analyses of infectious disease and other biological processes. Kitaake has a short generation time of approximately 9 wk, is easy to manipulate with classical genetic techniques, and has a remarkably small genome (450 Mb) compared with other monocotyledonous species (27). Kitaake is susceptible to *Xoo,* the causal agent of bacterial leaf blight disease, the most serious bacterial disease in Asia and Africa. This host–microbe interaction provides an attractive system for studies of infectious disease because both the host and bacterium are amenable to molecular genetic techniques (12). Studies of *Xanthomonas* have resulted in exciting discoveries, including the identification of transcription activator-like effectors (TALEs), the generation of TALE nucleases for gene editing (28–31), and the first medical application of genome editing: treatment of children with acute lymphoblastic leukemia (32).

Multiple races of *Xoo*, as well as rice cultivars with distinct *Xanthomonas* (*Xa*) resistance genes that confer resistance to specific races, have been characterized (33). In 1989, a new source of resistance was identified in the wild rice species *Oryza longistaminata* (34). Unlike other *Xa* genes reported at that time, this dominant locus conferred resistance to a broad spectrum of *Xoo* races (35, 36). Plant breeder and World Food Prize winner Gurdev Khush and colleagues at the International Rice Research Institute mapped this trait to chromosome 11 and named the locus *Xa21*.

At that time, positional cloning provided a promising method for isolation of genes that had been located on a genetic linkage map. This strategy consists of identifying DNA markers tightly linked to the gene of interest, isolating clones containing these markers from a genomic library, and complementing the recessive phenotype by transformation with candidate clones.

In 1992, Ronald and coworkers reported the genetic and physical mapping of the *Xa21* resistance locus using the nearly isogenic lines developed by Khush and colleagues. We identified three polymorphic DNA markers that were within 1.2 cM of *Xa21* on rice chromosome 11 and were physically linked to each other (37). These markers were used as starting points for a chromosome walk to the *Xa21* locus by Guoliang Wang and Wenyuan Song, postdoctoral researchers in the P.R. laboratory at the University of California, Davis.

## The Structure of the XA21 Immune Receptor

Wang, Song, and coworkers transformed the DNA fragments at the *Xa21* locus into a rice plant that is normally susceptible to bacterial infection (23). Fifty independently transformed rice plants, all containing a 9.6-kb DNA fragment, displayed high levels of resistance to *Xoo*. The sequence of the predicted protein within this fragment encoded an RLK with an LRR motif in the extracellular domain, a transmembrane domain, and an intracellular serine–threonine kinase domain, suggesting a role in cell-surface recognition of a pathogen ligand and subsequent activation of an intracellular defense response (Fig. 1).

The few plant RLKs that had been studied to date carried serine–threonine specificity in the kinase domain. One of these proteins, the *Brassica oleracea* S-receptor kinase (SRK) (38), had been shown by the group of June Nasrallah to mediate self-recognition between pollen and stigma during pollination. The biological functions of other plant RLKs that had been isolated at that time were unknown (39, 40).

These studies demonstrated that the plant RLK XA21 has a specific function, namely pathogen recognition and response. We further showed that although the *Xa21* locus consists of several tightly linked paralogs, a single gene product at the *Xa21* locus is sufficient to confer robust, broad-spectrum resistance (23). Its ability to protect against diverse races of the bacterium suggested that XA21 recognizes a conserved determinant present in every race of the pathogen.

## Similarity of Animal and Plant Immune Receptors

Subsequent discoveries in flies, humans, mice, and *Arabidopsis thaliana* revealed that animals and other plant species also carry membrane-anchored receptors with striking structural similarity to XA21 and that these receptors also play key roles in the immune response. For example, in 1996 the team of Jules Hoffman demonstrated that the *Drosophila* TOLL receptor is critical for resistance to fungal infection. TOLL-mediated perception of the fungus leads to the production of antimicrobial peptides that combat infection (41). Like XA21, TOLL carries LRRs in the extracellular domain. Furthermore, the associated TOLL kinase, called Pelle, falls into the same nonarginine aspartate (non-RD; see below) class of kinases as the XA21 kinase. In 1998, Bruce Beutler’s group isolated TOLL-like receptor 4 (TLR4) from mice (42). Like XA21 and TOLL, mouse TLR4 signals through a non-RD kinase, interleukin-1 receptor-associated kinase (IRAK) (Fig. 1). These studies indicated that, in animals, recognition of microbial molecules at the cell surface is mainly accomplished by the TLR family that also contains LRRs in the extracellular domain. TLRs activate both distinct and overlapping signaling pathways to induce a core set of proinflammatory and defense responses via associated non-RD kinases.

In 2000, Thomas Boller’s group isolated an *Arabidopsis* RLK that recognizes bacterial flagellin (43). FLAGELLIN SENSING 2 (FLS2) has a structure similar to XA21, with an LRR extracellular domain, a transmembrane domain, and a non-RD kinase integral to the receptor (Fig. 1). With the discovery in 2001 that TLR5 served as the animal receptor for flagellin (44), a clear, irrefutable picture emerged: Plants and animals use similar types of cell surface sensors to detect conserved microbial signatures, revealing an exciting convergence of plant and animal biology.

**Fig. 2.**
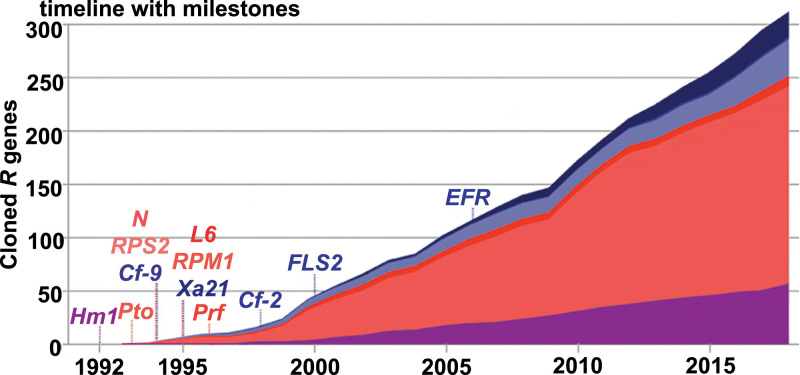
Thirty years of plant resistance gene cloning. The cloning of the first resistance gene was published in 1992. XA21 was isolated in 1995. The colors represent the proposed mechanism of resistance protein function. XA21 is representative of a class of cell-surface receptors that interact directly with their ligands, shown in dark blue. Light blue: cell surface receptors that interact indirectly with ligand (or interaction is unknown). Dark red: intracellular NBS-LRR receptors that interact directly with ligand. Light red: NBS-LRR receptors that interact indirectly with ligand (or interaction is unknown). Purple: other mechanisms (not all reproduced here). Of the 314 resistance genes examined in this 2018 study by Kourelis and van der Hoorn (45), only 128 have a proposed molecular mechanism. Modified from Kourelis and van der Hoorn (45), which is licensed under CC BY 4.0 (https://creativecommons.org/licenses/by/4.0/). In rice, there are hundreds of additional receptor kinases predicted by sequence analysis to function in immunity that have not yet been characterized (140).

During the past 30 y, the number of cloned resistance genes has steadily increased (45) (Fig. 2). The discovery of these receptors and characterization of their structures, ligands, and signaling cascades continues to reveal new mechanisms governing the plant innate immune response.

## RaxX, a Microbial Molecule Required for Activation of XA21-Mediated Immunity

**Fig. 3.**
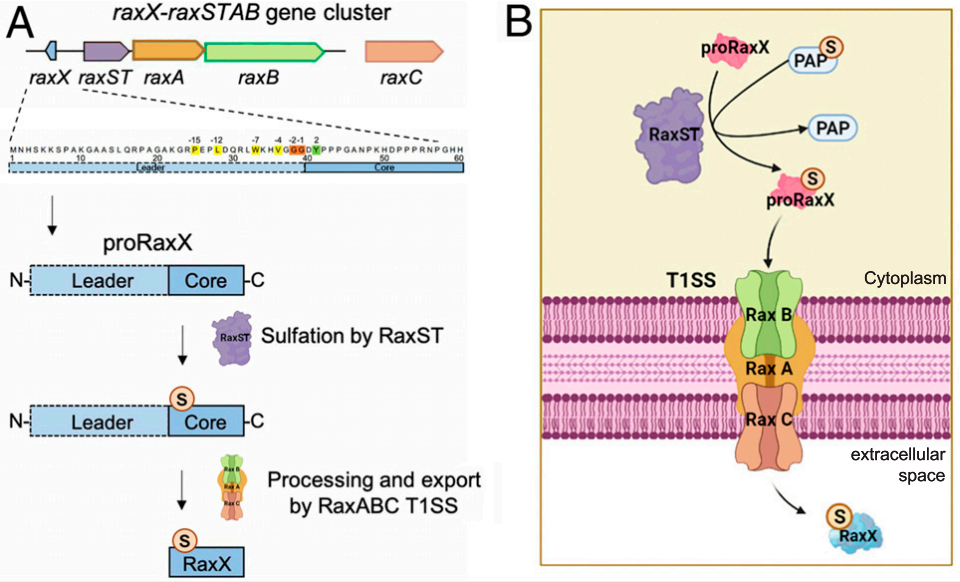
Biosynthetic pathway of RaxX, a tyrosine sulfated RiPP. (*A*) RaxX RiPP biosynthetic pathway. In general, the RiPP precursor (propeptide) and biosynthetic proteins are ribosomally synthesized. The core, which becomes the final RiPP product, is posttranslationally modified by enzyme(s) encoded in the same genomic region. Multiple posttranslational modifications can take place on a single propeptide. The N-terminal leader is enzymatically removed by a protease, and the modified core is exported by a transporter, releasing the mature bioactive RiPP. In the case of RaxX, proRaxX is ribosomally synthesized, and the core is sulfated by the sulfotransferase RaxST encoded upstream. The peptidase-containing transporter RaxB removes the N-terminal leader and transports the sulfated mature RaxX peptide through the T1SS composed of RaxB, the periplasmic adaptor protein RaxA, and the genetically unlinked outer membrane protein RaxC. (*B*) Cellular localization of RaxX and its biosynthetic proteins in *Xoo*.

The next goal of the P.R. laboratory was to identify the putative microbial molecule that triggers XA21-mediated immunity. In 2004, graduate student Francisco Goes da Silva identified and demonstrated that the *raxSTAB* gene cluster in the *Xoo* genome is required for activation of XA21-mediated immunity (46) (Fig. 3*A*). *raxST* encodes a tyrosyl-protein sulfotransferase (47), whereas *raxA* and *raxB* encode components of a bacterial type I secretion system (T1SS).

Analysis of the RaxB predicted protein was particularly informative. The *raxB* gene encodes a peptidase-containing ATP-binding cassette (ABC) transporter (48). The RaxB protein is most similar to a group of ABC transporters that secrete diverse peptides by cleaving their conserved N-terminal double glycine (GG)-leader sequence (49) (Fig. 3*B*). This analysis suggested that XA21 recognizes a T1SS-secreted GG-leader processed peptide. The presence of *raxST* in the operon suggested that the secreted peptide is sulfated on one or more tyrosyl residues. At that time, T1SS-secreted GG-leader peptides had no known role in the interaction of bacteria with their hosts.

Further support for the hypothesis that XA21 recognizes a sulfated peptide came from the report by Matsubayashi et al. in 2002 that the sulfated peptide phytosulfokine (PSK), which plays a key role in cellular dedifferentiation and proliferation in plants, binds an LRR-RLK (PSKR) (50).

Initial attempts in the P.R. laboratory to identify GG-leader peptides encoded in the *Xoo* genome as well as other approaches to identify the microbial ligand for XA21 failed (51). When postdoctoral fellow Rory Pruitt joined the laboratory, he started afresh and focused on the *raxSTAB* genomic region. Because GG-leader peptide genes typically are linked to genes for their secretory apparatus, Pruitt conducted a methodical genetic analysis of the *raxSTAB* region using insertions, deletions, and complementation tests. These experiments led to the discovery of a 60-amino-acid peptide named “RaxX,” carrying a predicted GG-leader that had not previously been annotated (Fig. 3*A*). Pruitt showed that deletion of the putative *raxX* gene allowed bacteria to escape detection by XA21 and cause disease in XA21 plants. Complementation analyses confirmed these results (52). Sequence analysis showed that field strains of *Xoo* that evade XA21-mediated immunity carry variations in the RaxX sequence (52). Together these results suggested that XA21 specifically recognized RaxX. Postdoctoral fellow Benjamin Schwessinger and others confirmed this hypothesis with experiments showing that sulfated RaxX alone (in the absence of the bacterium) is sufficient to activate XA21-mediated immune responses. In this paper, the team also showed that immunogenic activity mapped to the C terminus of the RaxX protein (52).

Several predictions from this initial study have been verified by subsequent work. Postdoctoral fellows Dee Dee Luu, Anna Joe, and others in the P.R. laboratory showed that the RaxX precursor peptide (proRaxX) is cleaved at the GG motif yielding a mature peptide and that proRaxX is processed and secreted by the RaxB peptidase-containing ABC transporter (53) (Fig. 3). These studies 1) established the predicted GG cleavage site in the proRaxX leader sequence, 2) found the predicted sTyr-containing mature RaxX peptide in the extracellular milieu, and 3) documented the predicted peptidase and secretion functions for the RaxB protein. With important contributions from our collaborators in Youssef Belkhadir’s laboratory, we also demonstrated the predicted high-affinity binding of sulfated RaxX directly to the XA21 LRR domain (53).

In addition to identifying the ligand for XA21, our studies revealed that RaxX is the first identified prokaryotic member of a previously unclassified and understudied group of tyrosine sulfated ribosomally synthesized and posttranslationally modified peptides (RiPPs) (53, 54). RiPPs are structurally and functionally diverse natural products, with many displaying potent therapeutic activity (54). RaxX represents one group that has not been well-studied or formally categorized as RiPPs-tyrosine sulfated (sTyr) peptides. The role of this class of RiPPs in microbial, plant, and animal physiology is a new field of research ripe for exploration.

## RaxX Regulation

Once we identified RaxX as both the ligand for XA21 and as the substrate for the associated RaxST-RaxBA posttranslational modifications, we turned to understanding how *raxX-raxSTAB* gene expression is regulated.

It has long been known that many *Xanthomonas* species induce a hypersensitive reaction on resistant plants and disease symptoms on susceptible plants upon infection, collectively named hypersensitive reaction and pathogenicity (Hrp) phenotypes (55). These phenotypes depend upon *Xanthomonas* outer protein (*xop*) genes (56), which encode effector proteins that disrupt numerous aspects of host cell function and signaling (57), and *hrp* genes (58), which encode a type III secretion system (T3SS) that translocates these effector proteins into the host cytoplasm (57). *hrp* and *xop* gene expression is induced *in planta* (59) and, for *Xoo*, in the xylose-containing XOM2 defined medium (60). This plant-inducible expression requires the DNA-binding transcription activator HrpX (61, 62). In most cases, HrpX-dependent transcription requires a PIP (plant-inducible promoter) box sequence, which forms the binding site for HrpX (63–70). HrpX synthesis is governed by a complex regulatory network that converges on the response regulator HrpG, which activates *hrpX* transcription (71–73). Thus, the HrpX protein directly activates *hrp* and *xop* gene transcription initiation. As a global regulator of pathogenicity, HrpX also regulates a diverse array of other microbial “virulence factors” that facilitate infection (71, 73, 74).

**Fig. 4.**
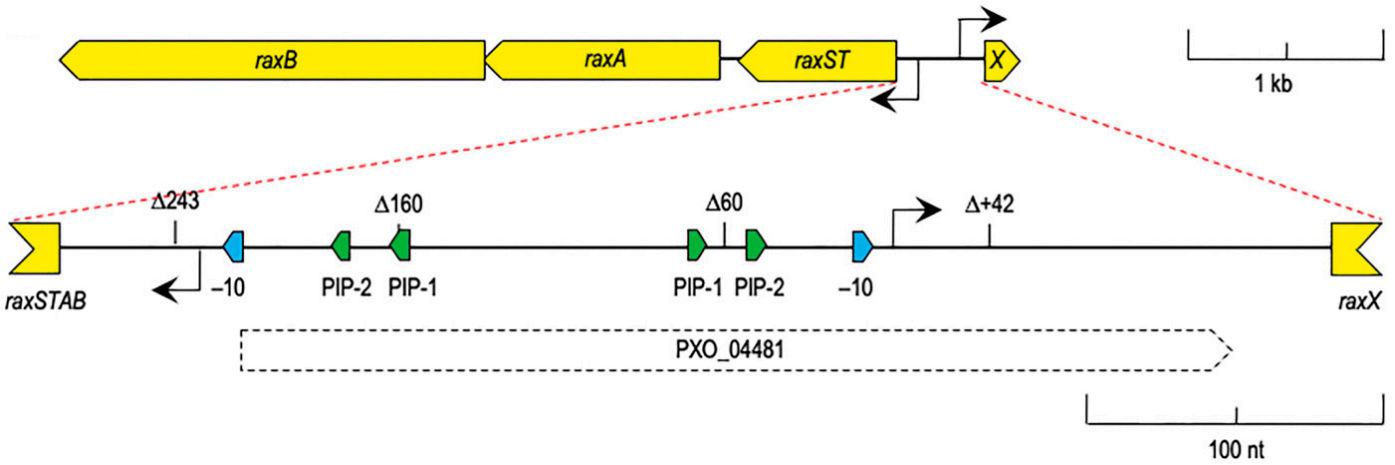
The *raxX-raxSTAB* divergent transcription control region. The *raxX-raxSTAB* gene cluster includes the 431 bp control region (*Inset*) between the *raxST* and *raxX* initiation codons. Relative locations are shown for the –10 box sequences (blue), PIP box sequences (green), and predicted transcription initiation sites (arrows). Drawn to scale.

Based on knowledge of the importance of HrpX in *Xanthomonas* pathogenicity, we hypothesized that HrpX may also regulate *raxXSTAB*. Indeed, investigations by postdoctoral fellow Joe revealed that both *Xoo raxX* and *raxST* are activated by HrpX during growth *in planta* and in XOM2 medium (75). Joe further identified PIP box promoter motifs preceding the transcription start site of each gene (Fig. 4).

These findings expand the set of genes regulated by HrpX to include RaxX, secreted by a T1SS, in addition to previously identified T2SS- and T3SS-secreted factors, and support a role for RaxX as a virulence factor activated upon entry into plants. Indeed, the *raxX-raxSTAB* gene cluster is maintained in many Xanthomonads (76), indicating that RaxX provides fitness benefits to diverse *Xanthomonas* species, presumably during interactions with their wide range of monocot and dicot hosts. This hypothesis is supported by in vivo data showing that *Xoo* strains lacking the *raxX* or *raxST* genes are compromised in virulence (52, 77).

## RaxX Mimics a Plant Peptide Hormone

Although the precise role of RaxX in *Xoo* biology is not yet known, the sequence of RaxX and its effect on plant root development provide some clues to its possible function (77).

**Fig. 5.**
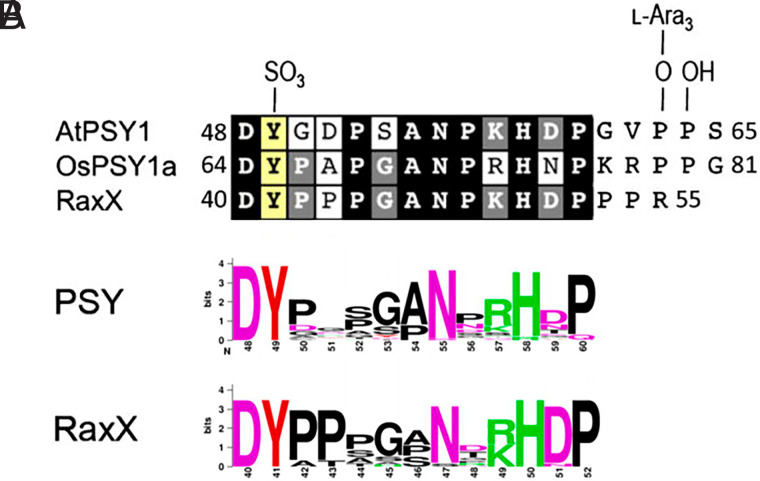
A microbially derived tyrosine‐sulfated peptide mimics a plant peptide hormone. Sequence similarity of RaxX and plant peptides containing sulfated tyrosine (PSYs). (*A*) The mature 18-amino-acid AtPSY1 (amino acids 48 to 65 of the AtPSY1 precursor protein) and a synthetic PSY-like repeat from OsPSY1 (amino acids 64 to 81 of the OsPSY1 precursor protein) were aligned with the sequence of RaxX peptides from *Xoo* strain PXO99. The numbers adjacent to the sequence indicate the amino acid positions of the terminal peptide residues within the predicted precursor protein. Endogenous AtPSY1 has three posttranslationally modified residues, which are shown at the top of alignment: a sulfotyrosine and two hydroxyprolines. The first hydroxyproline is further modified by a chain of three l-arabinose residues (l-Ara_3_). Residues in the black box are identical in all three sequences. The gray boxes indicate a conserved residue in two sequences among AtPSY1, OsPSY1a, and RaxX. The sulfated tyrosine is marked in a yellow box. (*B*) Sequence logos depicting the amino acid composition in the conserved 13-amino-acid region of RaxX and PSY proteins. The logos were generated from 34 PSY orthologs and 17 nonredundant RaxX13 sequences. Modified with permission from Pruitt et al. (77).

In 2015, Weiguo Zhang, a postdoctoral fellow in the P.R. laboratory, treated *Arabidopsis* seedlings with synthetic sulfated C-terminal fragment of RaxX and observed that the sulfated peptide enhanced root growth (77). Subsequent experiments by Joe showed that sulfated RaxX also enhances root growth in rice (77). Sequence analysis of diverse plant genomes by postdoctoral fellows Pruitt and Schwessinger led to the discovery that the C terminus of proRaxX is similar to the peptide hormone PSY (Plant peptide containing Sulfated tYrosine) (52, 77) (Fig. 5). *Arabidopsis* PSY1 (AtPSY1), the best-characterized member of the plant PSY peptide family, promotes cellular proliferation and expansion (78). AtPSY1 is an 18-amino-acid glycopeptide with a single sulfotyrosine residue that is processed from a 75-amino-acid precursor, secreted, and promotes root elongation primarily through regulation of cell size (78).

**Fig. 6.**
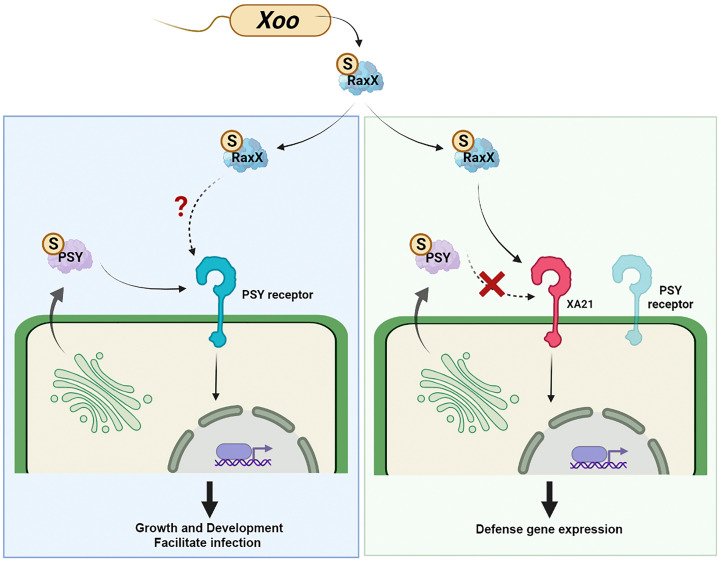
Model for XA21 immune function. Based on the studies demonstrating the growth-stimulating activity of PSY and our findings in rice and *Arabidopsis*, we hypothesized that *Xanthomonas* produces, sulfates, and secretes RaxX to mimic the activity of PSY peptides. Unlike RaxX, PSY peptides do not activate XA21-mediated immunity. We hypothesize that *O. longistaminata* evolved XA21 to specifically recognize RaxX. Consequently, rice plants carrying XA21 are able to launch a defense response against the pathogen but not the highly similar endogenous PSY peptide hormones, which are predicted to be necessary for normal growth and development. The hypothesis that RaxX is a mimic of PSY is well-supported by the high level of sequence similarity, the tyrosine sulfation status of RaxX and PSY peptides, and the similar growth-promoting activities of both peptides. Thus, XA21 is a highly selective immune receptor capable of specifically recognizing the bacterial mimic.

Based on these studies demonstrating the growth-stimulating activity of PSY, and our findings in rice and *Arabidopsis*, we hypothesized that *Xoo* produces, sulfates, and secretes RaxX to mimic the activity of PSY peptides (46, 52, 77) (Fig. 6). Unlike RaxX, PSY peptides do not activate XA21-mediated immunity (77). We hypothesize that in *O. longistaminata* XA21 evolved to specifically recognize RaxX. Consequently, rice plants carrying XA21 can launch a defense response against the pathogen but not the highly similar endogenous PSY peptide hormones, which are predicted to be necessary for normal growth and development. The hypothesis that RaxX is a mimic of PSY is well-supported by the high level of sequence similarity, the tyrosine sulfation status of RaxX and PSY peptides, and the comparable growth-promoting activities of both peptides (52, 77, 78). Thus, XA21 is a highly selective immune receptor capable of specifically recognizing the bacterial mimic.

Diverse microbes have been demonstrated to employ molecular mimics to hijack the plants’ endogenous systems and reprogram the host environment to facilitate pathogen infection (79–82). For example, nematodes produce peptides similar to plant CLAVATA3/Embryo-Surrounding Region (CLE) peptides (82), which regulate shoot meristem differentiation, root growth, and vascular development. Nematode CLEs are secreted into plant tissues where they induce specific host cells to differentiate into feeding cells that benefit the parasite (83–85). Based on this example and our results, we hypothesize that *Xoo* employs RaxX in a similar manner. *Xoo* is a biotrophic pathogen and thus requires living host tissues, which ensures a prolonged supply of carbon and other nutrients necessary for bacterial survival. The ability of *Xoo* to utilize RaxX to promote host growth would thus benefit a biotroph (86, 87).

## Terminology

In this review, we classify XA21 as a cell-surface “immune receptor.” We prefer this broad term rather than “Resistance (R)” gene or “Pattern recognition receptor (PRR)” because XA21 shares properties with both these classifications. For example, the term PRR is typically used to refer to proteins that recognize conserved pathogen-associated molecular patterns (PAMPs) and transduce “PAMP-triggered immunity (PTI).” XA21 shares many similarities with PRRs: All carry non-RD kinase domains, associate with somatic embryogenesis receptor kinases (SERKs) such as BAK1/OsSERK2 (88), and activate rapid reactive oxygen species responses after treatment with their microbial ligands (89, 90). The term “R gene product” is often used to denote intracellular NBS-LRR type genes that transduce “Effector-triggered immunity (ETI).” Like *Xa21*, many genes for NBS-LRR proteins confer a race-specific and robust resistance response.

The microbial molecule RaxX shares properties with PAMPs and with microbial effectors. Like many PAMPs, RaxX is present outside the bacterial cell where it can interact with PRRs. The epitope regions of both flg22 and RaxX display sequence divergence to avoid detection by the host receptor (52, 76, 91). RaxX also shares similarities with microbial effectors, which are targeted to plant cells and confer benefits to the pathogen upon entry into the plant host (92). Similar to T3SS-secreted effectors, RaxX synthesis is regulated by HrpX (75). Also like these effectors, RaxX possibly manipulates plant signaling to promote bacterial infection and symptom progression.

The PTI/ETI distinction as originally proposed has blurred with the discovery and characterization of more receptor/ligand pairs and their downstream partners, which have revealed the overlap between resistance mechanisms (20, 52, 93). Similarly, as more microbial factors are identified that do not neatly fit into the PAMP vs. effector dichotomy, such classifications may lose relevance (91, 94–97).

## Tyrosine Sulfation Mediates Extracellular Protein–Protein Interactions

Both RaxX and PSY1 require tyrosine sulfation for full activity. Tyrosine sulfation is an important posttranslational modification for certain extracellular protein–protein interactions. Plants and animals employ tyrosine-sulfated proteins to regulate growth, development, immunity, and other biological processes. In animals, this includes coagulation, leukocyte adhesion, HIV entry, and chemokine signaling (98–100). For example, in humans, sulfation of the C–C chemokine receptor type 5 (CCR5) is critical for binding of the envelope glycoprotein gp120 of HIV (101). Tyrosine sulfation also plays important roles in malaria–cell interactions (102), the control of blood clotting (hemostasis) (103–106), inhibition of the host immune response by the highly virulent methicillin-resistant bacterium *Staphylococcus aureus* (107), immune cell signaling and migration (108, 109), peptide hormone signaling (110–112), and pathogen perception and entry (113). The recent discoveries of a potent HIV entry inhibitor (114) and a new class of thrombin inhibitors (115) demonstrate the relevance of studies of sulfation biology to medicine.

**Fig. 7.**
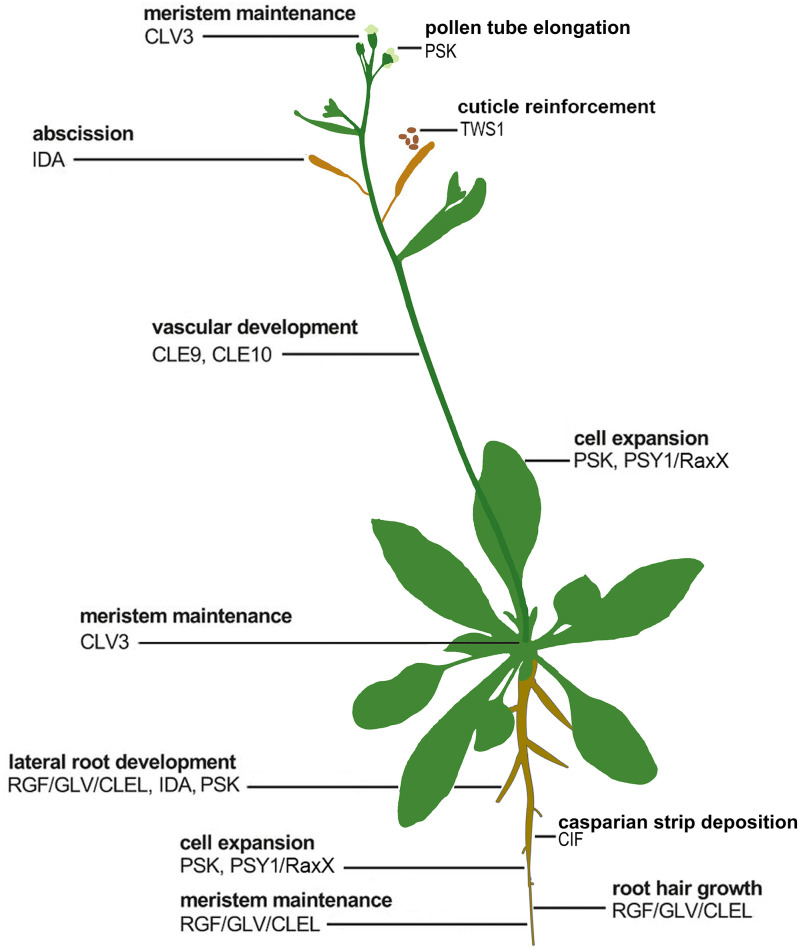
Functional diversity of posttranslationally modified peptides. Posttranslationally modified peptides are characterized by specific posttranslational modifications, such as Pro hydroxylation, glycosylation, and tyrosine sulfation; and are released from longer precursors known as propeptides. These peptides participate in the control of a wide range of biological activities in plant development, including the establishment of cell identity or specific cellular structures. The activity of these peptides relies on their expression pattern and their ability to interact with their specific receptors. Modified from Tavormina et al. (124), which is licensed under CC BY 4.0 (https://creativecommons.org/licenses/by/4.0/).

During the past several years, there have been numerous exciting discoveries of plant receptors that recognize sulfated peptides, as recently reviewed (116). In addition to PSY, plants produce four other classes of tyrosine sulfated peptides that bind LRR receptor kinases: phytosulfokine (PSK) (50, 117), root meristem growth factor (RGF) (118), Casparian strip integrity factor (CIF) (119–122), and twisted seed 1 (TWS1), which shares sequence similarity to CIF (123). Like PSY, PSK, RGF, CIF, and TWS1 are processed, secreted, and play roles in a variety of processes involved in the regulation of plant growth and development (117–120, 123, 124) (Fig. 7).

Despite recent progress, our knowledge of the composition of the sulfated complexes controlling these responses remains limited and the structural determinants have only been elucidated for a limited set of examples (110, 115, 125).

### LRR Receptors and Coreceptors (RLKs and RLPs) Mediate Plant Immunity and Development.

Since the characterization of the first RLKs with known function, rice XA21 and *B. oleracea* SRK (38), there has been an explosion of research into the investigation of RLK phylogeny and function.

**Fig. 8.**
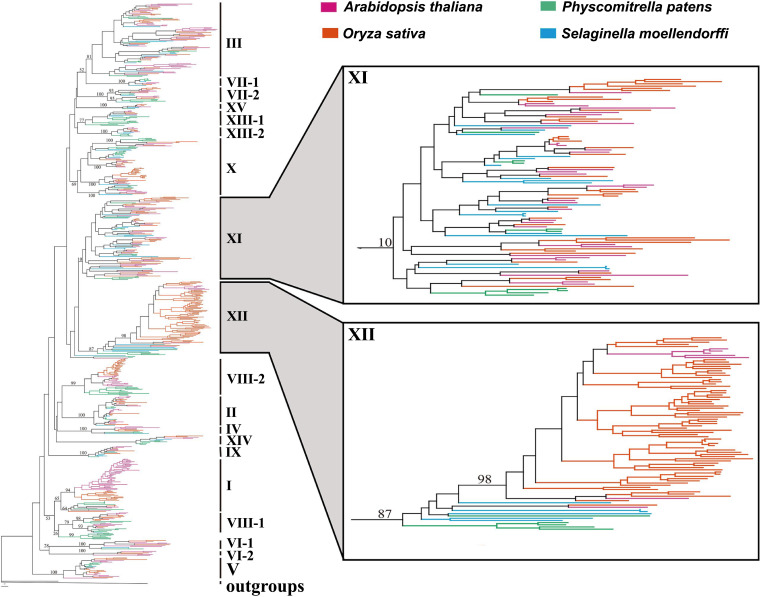
Phylogenetic tree of LRR-RLK genes comparing rice (*O. sativa*), *A. thaliana*, moss (*Physcomitrium patens*), and lycophyte (*Selaginella moellendorffii*). Phylogenetic tree was constructed by Liu et al. (128) based on comparison of the kinase domain amino acid sequences in each LRR-RLK subfamily with sequences from rice, *Arabidopsis*, *P. patens*, and *S*. *moellendorffii*. The figure highlights the LRR-RLK XI and XII subfamilies. The XI subfamily includes many receptors, such as CLV1 and RGI1,2,3, that recognize plant produced peptides. The XII subfamily includes the rice receptor XA21 and several *Arabidopsis* immune receptors that recognize microbial peptides, such as FLS2 which recognizes bacterial flagellin and EFR which recognizes bacterial EF-Tu. Above each branch bootstrap values from the phylogenetic analysis are shown with subfamily labels on the right of each cluster. Pink indicates *Arabidopsis*, red indicates rice, green indicates *P. patens*, and blue indicates *S. moellendorffii*. Modified from Liu et al. (128), which is licensed under CC BY 4.0 (https://creativecommons.org/licenses/by/4.0/).

For example, in 2001 and 2004, Shiu and team categorized the LRR-RLKs of *Arabidopsis* and rice (126, 127). They found that in contrast to RLKs involved in development, those involved in defense have undergone many duplication events since the *Arabidopsis*–rice split. These findings led them to hypothesize that defense/resistance-related genes account for most of the recent expansion of the RLK/Pelle family. The RLK subfamily that most differentially expanded between rice and *Arabidopsis* was the LRR-XII subfamily, with >150 rice genes compared to only 6 in *Arabidopsis*. This subfamily includes XA21 (23) and *Arabidopsis* immune receptors FLS2 and EFR (43). In 2017 Liu et al. further analyzed the LRR-RLK gene family by comparing previously described LRR-RLK sequences in *Arabidopsis* and rice to other divergent plant species such as algae, moss, and lycophytes (128). Although they did not identify any LRR-RLK genes in any algae species, they did identify LRR-RLK genes in *Physcomitrium patens* (moss, previously *Physcomitrella patens*) and *Selaginella moellendorffii* (lycophyte). These LRR-RLK genes clearly separate into 19 distinct subfamilies after comparing conserved LRR kinase domain sequences with sequences from each of these species (Fig. 8), supporting previous phylogenetic analyses of *Arabidopsis* LRR-RLK genes (126). Additionally, evidence accumulated over the last 30 y indicates that the LRR receptor subfamily XI recognizes intrinsic peptides (such as plant peptide hormones), whereas receptor subfamily XII recognizes extrinsic peptides (such as microbial molecules) (Fig. 8).

Many research groups have probed the function of RLKs, RLPs, and their predicted ligands in diverse species (Fig. 7) (129, 130). For example, in 1997 Clark et al. showed that CLAVATA 1 encodes a receptor kinase that mediates meristem development and recognizes CLV3/ENDOSPERM SURROUNDING REGION (ESR)-related (CLE) peptides (131). Other important discoveries include the identification of the RLK brassinosteroid insensitive 1 (BRI1) that is required for Brassinosteroids (BR) perception to regulate development (131); the closely related receptors HAESA (HAE, formerly named RLK5) and HAESA-LIKE 2 (HSL2) that recognize the peptide ligand INFLORESCENCE DEFICIENT IN ABSCISSION (IDA) to regulate floral organ abscission (132–134); and the RGF1-INSENSITIVE 1–5 (RGI1–5) (RGI/RGF) receptors; RGI1 and RGI2 have been shown to regulate root meristematic activity via interaction with RGF peptides (110, 135) (Fig. 7).

In 2002, the Chory laboratory showed that BRI1-associated receptor kinase 1 (BAK1) forms a protein complex with BRI1 during perception of BRs (136, 137). In 2007, two laboratories showed that FLS2 forms a heterodimer with BAK1 in the presence of flagellin (138, 139). *bak1* mutant plants are impaired in responsiveness to flagellin. These studies showed for the first time that a single coreceptor, BAK1, could function in both plant development and immune signaling.

In 2006, postdoctoral fellow Chris Dardick in the P.R. laboratory studied a small functional class of kinases termed non-RD kinases that do not autophosphorylate the activation loop, as is typical for most kinases (140). XA21, EFR, and FLS2 belong to this class of non-RD kinases. A survey of the yeast, fly, worm, human, *Arabidopsis*, and rice kinomes (3,723 kinases) revealed that despite the small number of non-RD kinases in these genomes (9 to 29%), 12 of 15 kinases known or predicted to function in immune signaling fall into the non-RD class. In other words, kinases known or predicted to function in recognition of conserved microbial signatures fall into the non-RD class or associate with a non-RD kinase. These data suggest that kinases associated with immune function can largely be predicted by the lack of a single conserved residue.

Some RD kinases can participate in both developmental and immune responses. For example, BAK1 is an RD RLK coreceptor that interacts with diverse RLKs as described above. Similarly, OsSERK2 (the rice ortholog of *Arabidopsis* BAK1) serves as a coreceptor for XA21-mediated immunity (88). Another example is *Arabidopsis* SOBIR1 (Suppressor Of BIR1-1), an RLK with an RD domain that functions in diverse immune responses mediated by RLPs that lack the kinase signaling domain (141). SOBIR1 interacts with RLP23 to facilitate immune response to *Pst* (142). The RLP23/SOBIR1 complex, which also requires BAK1 coreceptor for function, is particularly interesting because the output requires proteins that were previously shown to be essential to immunity mediated by the NBS-LRR intracellular class of receptors (94). In addition to *Arabidopsis* RLP23, SOBIR is essential for the function of other RLPs, such as Cf-4 in tomato, indicating its broad involvement in RLP-mediated immunity as a coreceptor (94, 141).

The RGI family is of particular interest for investigations into the possible biological function of the non-RD motif. This family contains five members that belong to both the non-RD and RD kinase classes (*SI Appendix*) (143). For example, RGI1 is an RD kinase that associates with RGF1 to regulate root development (143). RGI3, a non-RD kinase, forms an flg22-induced complex with the flg22-receptor FLAGELLIN SENSITIVE 2, suggesting that RGI3 is part of an activated cell surface immune receptor signaling complex (144). Additional research on the biological significance of the RGI-mediated responses may shed mechanistic insight into the association of non-RD with immune function.

In rice, the RLP XA21D, one of the XA21 paralogs described above, confers a partial resistance response to *Xoo* (145). The coreceptor for XA21D-mediated immunity has not yet been identified. Unlike RLPs that interact with SOBIR1, which all have a membrane anchor, XA21D is predicted to be a secreted RLP lacking a transmembrane domain, similar to the secreted *S*-locus glycoprotein (SLG) that mediates the specificity of pollen–stigma interactions (146).

Despite much progress since the discovery and characterization of XA21, XA21D, and other plant RLKs and RLPs, much remains to be learned about how these ligand/receptor pairs function with other coreceptors to exert their developmental effects and how they interact with closely related defense signaling pathways. The majority of the RKs identified in plants have no known function and most of their signaling partners remain unknown. Similarly, most RLPs (ca. 56 in *Arabidopsis* ecotype Col-0; at least 90 in rice) remain uncharacterized with unknown functions (147–149).

Another poorly understood aspect of these receptors is how their tissue-specific expression and/or their interaction with different members of a ligand family may affect their functions. An excellent example of this complexity is the demonstration that the CLE9/10 peptide hormone regulates two different developmental processes in *Arabidopsis* through two distinct receptor systems (150). CLE9/10 regulates stomatal lineage cell division through the HSL class of receptor kinases but regulates periclinal cell division of xylem precursor cells through the BARELY NO MERISTEM (BAM) class receptor kinases. Both HSL1 and BAM1 bind to CLE9/10, but only HSL1 recruits SERKs as coreceptors, suggesting different signaling modes for these receptor systems (150).

The P.R. laboratory is particularly interested in how receptors distinguish the related RaxX and PSY peptides. Based on the sequence similarity and shared function in root growth promotion, we hypothesize that PSY1 and RaxX target a common cognate plant receptor (Fig. 6). The LRR-RLK At1g72300 was originally hypothesized to serve as the receptor for AtPSY1 based on the observation that the root length was not increased by exogenous AtPSY1 treatment in an At1g72300 mutant (78). However, the At1g72300 mutant line still partially responds to AtPSY1 treatment in proton efflux experiments (151) and transcriptomics analysis revealed that many AtPSY1-regulated genes are regulated independently of At1g72300 (152). Furthermore, we found that RaxX and AtPSY1 still promote root growth in the absence of At1g72300 (77). Collectively, these findings indicate that At1g72300 is not the receptor for PSY peptides or that it is not the only receptor.

We hypothesize that the as-of-yet-unidentified PSY receptor(s) regulate different developmental processes through multiple PSY peptides in a tissue-specific manner, as was shown for the HSL1 and BAM receptors (150). Such a model would explain how PSY can exert a robust effect on root development in response to PSY treatment, whereas *Xoo,* a xylem pathogen, likely infects other tissues. For example, xylem parenchyma cells may express the same or a different PSY receptor that could account for RaxX function in virulence (Fig. 6). Growth and immunity are highly interlinked processes, as reflected by increasing reports demonstrating cross-talk between regulatory genes involved in the control of both processes (153–155). These observations suggest that RaxX, acting as a mimic of a growth-promoting hormone, may modify the plant developmental process in a way that would favor bacterial infection (156). Given that XA21 specifically recognizes RaxX peptides (77), we hypothesize that the XA21 immune receptor evolved after the PSY receptor to recognize this mimic and limit bacterial infection (Fig. 6).

Isolation and characterization of the putative PSY receptor (and coreceptor) followed by structural and functional studies comparing RaxX-XA21 and RaxX with the putative PSY receptor or PSY/PSY receptor complexes will provide insight into the mechanisms governing ligand recognition and will help us to understand how PSY and RaxX peptides are perceived in plants to induce root growth, facilitate infection, and trigger the immune response.

## Conclusion

In a classic evolutionary arms race, both the pathogen and host develop and deploy an arsenal of strategies to infect or resist their partner. Pathogens secrete an array of molecular factors designed to manipulate host biology and suppress the immune response. In turn, plants have developed a set of immune receptors that recognize these molecules or their activities and launch mechanisms to destroy the pathogen, which the pathogen then tries to counter. Decades of work on the rice immune receptor XA21-RaxX system has led to valuable insights into the molecular genetic basis of this evolutionary arms race. However, gaps remain in understanding the physiological function of pathogen-secreted factors, the molecular and structural requirements for their interaction with cell surface plant immune receptor complexes, the mechanistic significance of the non-RD motif in these receptors, and transduction of downstream immune signaling. A more complete picture of pathogen–plant interactions at the cell surface will help us tip the balance in the plant host’s favor.

## Data Availability

All study data are included in the article and/or *SI Appendix*.
